# La maladie de Bowen: un carcinome épidermoïde rare ou sous diagnostiqué

**DOI:** 10.11604/pamj.2014.17.251.4252

**Published:** 2014-04-07

**Authors:** Maha Mael-ainin, Badredine Hassam

**Affiliations:** 1 Service de Dermatologie, CHU Ibn Sina, Université Mohamed V, Souissi, Rabat, Maroc

**Keywords:** La maladie de Bowen, carcinome épidermoïde, Bowen's disease, squamous cell carcinoma

## Image en medicine

La maladie de Bowen correspond à un carcinome malpighien intra-épithélial. Il s'agit d'une forme in situ du carcinome épidermoïde cutané. Son incidence est rare ou sous estimée. La maladie de Bowen survient chez l'adulte avec un pic dans la septième décennie et une prédilection féminine. Les lésions sont uniques ou multiples, de siège ubiquitaire intéressant les zones photo-exposées ou couvertes. L'atteinte cutanée est la plus fréquente, les localisations muqueuses ou unguéales sont possibles. Au niveau cutané, ce carcinome prend l'aspect d'une plaque érythémateuse légèrement infiltrée recouverte de squames et de croûtes. Au niveau muqueux, les lésions peuvent être pigmentées, érythroplasiques ou leucoplasiques. L'atteinte unguéale se manifeste généralement sous forme d'une érythronychie longitudinale localisée. L'histologie révèle des anomalies de maturations, une perte de la polarité des cellules malpighiennes avec parfois des cellules monstrueuses aux noyaux volumineux et hyper chromatiques. Les lésions siègent au niveau épidermique sans franchissement de la membrane basale. Le pronostic de la maladie de Bowen est bon. Le risque de progression en carcinome invasif est de l'ordre de 3 à 20% des cas. Le traitement de choix repose sur l'exérèse chirurgicale précoce et complète avec des marges latérales de 5mm. Mme E.R, âgée de 67 ans, sans antécédents pathologiques, présentait depuis deux ans une plaque érythémato-croûteuse fixe, légèrement infiltrée siégeant en regard de l'hypochondre gauche, l'histologie était compatible avec une maladie de Bowen. L'exérèse chirurgicale était complète, la membrane basale était respectée, il n'y avait pas de micro-invasions. L’évolution était favorable sans récidive avec un recul de trois ans. Diagnostic retenue: Maladie de Bowen

**Figure 1:**
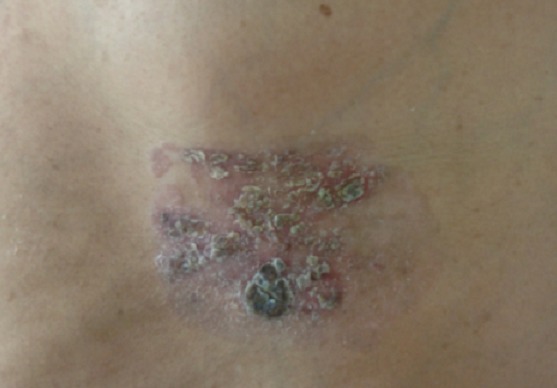
Plaque érythémato-croûteuse en regard de l'hypochondre gauche

